# Effects of a hydropower project on a high‐value Asian elephant population

**DOI:** 10.1002/ece3.10353

**Published:** 2023-07-25

**Authors:** Kris Budd, Daophone Suddychan, Martin Tyson, Camille N. Z. Coudrat, Alex McWilliam, Christopher D. Hallam, Arlyne Johnson, Lori S. Eggert

**Affiliations:** ^1^ Division of Biological Sciences University of Missouri Columbia Missouri USA; ^2^ Nam Theun 2 Power Company Limited (NTPC) Vientiane Lao PDR; ^3^ Biological Consultant Poolewe UK; ^4^ Association Anoulak Nakai District Lao PDR; ^5^ International Union for Conservation of Nature (IUCN) Bangkok Thailand; ^6^ School of Bioscience University of Melbourne Melbourne Victoria Australia; ^7^ Foundations of Success Bethesda Maryland USA; ^8^ Nelson Institute for Environmental Studies University of Wisconsin – Madison Madison Wisconsin USA

**Keywords:** elephants, habitat loss, human‐elephant conflict, hydroelectric dam, hydropower

## Abstract

Habitat loss and fragmentation are leading contributors to the endangered status of species. In 2006, the Nakai Plateau contained the largest known Asian elephant (*Elephas maximus*) population in the Lao People's Democratic Republic (Lao PDR), and the population was among those with the highest genetic diversity reported for Asian elephants. In 2008, completion of the Nam Theun 2 hydroelectric dam inundated much of the Plateau, resulting in the loss of 40% of elephant habitat. We studied elephant presence, movements, and the incidence of human–elephant conflict (HEC) on the Nakai Plateau and surrounding areas from 2004 to 2020, before and for 12 years after dam completion. To examine contemporary population dynamics in the Nakai elephants, we used genetic sampling to compare minimum population numbers, demography, and levels of genetic diversity from the wet and dry seasons in 2018/2019, 10 years after dam completion, with those reported in a pre‐dam‐completion genetic survey. After dam completion, we found a major increase in HEC locally and the creation of new, serious, and persistent HEC problems as far as 100 km away. While we were unable to compare estimated population sizes before and after dam completion, our data revealed a decrease in genetic diversity, a male‐biased sex ratio, and evidence of dispersal from the Plateau by breeding‐age females. Our results raise concerns about the long‐term viability of this important population as well as that of other species in this region. Given that hydropower projects are of economic importance throughout Laos and elsewhere in southeast Asia, this study has important implications for understanding and mitigating their impact.

## Graphical abstract

We studied elephant presence, movements, and the incidence of human‐elephant conflict (HEC) on the Nakai Plateau, Lao PDR, and surrounding areas before and for 12 years after the completion of the Nam Theun 2 hydroelectric dam. We also used genetic data to compare the population 10 years after dam completion to data from a pre‐dam completion survey. Our results demonstrate a major increase in HEC both locally and as far as 100 km away from the Plateau during the study period, a decrease in genetic diversity, a shift in the sex ratio, and an increase in the proportion of subadult males in the population. Our results provide insights into not only the direct local impacts of habitat loss and fragmentation on elephant populations, but also the longer term landscape‐level effects.

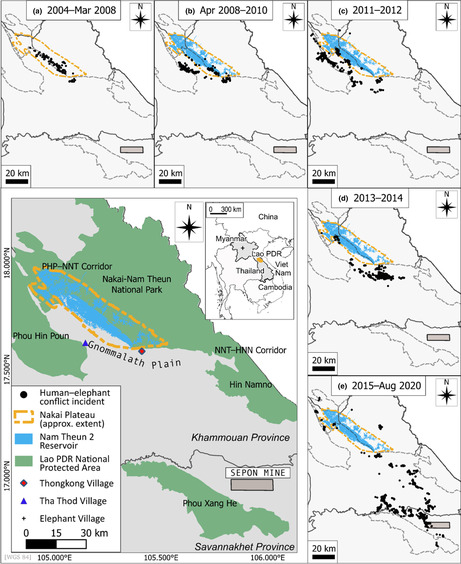

## 1 INTRODUCTION

Development activities such as roads, dams, and other infrastructure projects can result in the loss and fragmentation of wildlife habitat, block migration routes, and facilitate poaching of wild animals, including elephants (Choudhury, 2004). The Asian elephant (*Elephas maximus*) is listed as Endangered in the IUCN Red List of Threatened Species (Williams et al., 2020) and is included in CITES appendix I. It is threatened by habitat loss/transformation and fragmentation, poaching, and removals from the wild, both legal and illegal (Leimgruber et al., 2003; McWilliam et al., 2010; Sukumar, 1989). As habitat becomes less suitable due to decreased area and/or increased fragmentation, it becomes less able to support a viable population over the long term (Leimgruber et al., 2003). In addition, although elephants and other species may remain in an area following habitat transformation, doing so can place them and nearby human populations at extreme risk of conflict (Kushwaha & Hazarika, 2004).

In areas where human and elephant populations overlap, crop raiding by elephants is the main source of conflict (Cabral de Mel et al., 2022; IUCN, 2023). Elephants trample and feed in cultivated fields, particularly at night, resulting in losses of crops and damage to structures that store harvested grains, losses that are particularly acute for low‐income subsistence farmers whose fields are near forests and protected areas. In addition to severe economic losses, human–elephant conflict (HEC) results in an alarming number of deaths of both elephants and humans. For instance, between 2015 and 2018 an average of 124 elephants and 571 humans were killed annually in India (Cabral de Mel et al., 2022). Despite efforts by local governments and non‐governmental organizations to prevent and mitigate the effects of HEC (Shaffer et al., 2019), it breeds hostility from local populations and erodes support for elephant conservation.

The Lao People's Democratic Republic (Lao PDR, hereafter referred to as Laos) historically contained extensive elephant habitat and travel corridors (Khounboline, 2011). While elephant populations have declined, as they have across much of southeast Asia, central Laos, and especially the Nakai Plateau area, was thought to contain one of the two most important elephant populations in the country (Duckworth & Hedges, 1998). More generally, the Nakai–Nam Theun National Park (NNT NP), spanning the north‐east half of the Nakai Plateau into the Annamite Mountains up to the Lao–Vietnam border, is considered a biodiversity hotspot (Myers et al., 2000).

Over recent decades, the countries of Southeast Asia have experienced rapid economic and population growth, leading to increases in energy needs (Sakti et al., 2023).

Between 2005 and 2008, the Nam Theun 2 (NT2) hydroelectric dam, one of the largest dam projects in Southeast Asia, was constructed on the Nam Theun River. To assess the conservation significance of the elephant population on the Nakai Plateau and inform wildlife management strategies to help mitigate the impact of the NT2 project prior to the creation of the reservoir on the plateau, a dung‐count‐based survey and a simultaneous genetic capture–mark–recapture (CMR) study were conducted from February to May 2006 (Ahlering, Hedges, et al., 2011; Hedges et al., 2013). These studies estimated the Nakai population at 141 individuals (95% CI = [95, 208]) using the dung‐count method and 132 (95% CI = [120, 149]) using the genetic CMR method, with 102 unique genotypes identified (Ahlering, Hedges, et al., 2011). The studies found that the Nakai population had a combination of high genetic diversity, largely intact social structure, and relatively large size, all of which identified it as having high conservation value (Ahlering, Hedges, et al., 2011; Hedges et al., 2013). In subsequent comparisons of the results of the 2006 Nakai study with elephant populations elsewhere in Asia, Ahlering et al. (2020) reiterated the importance of the high levels of genetic diversity found in the Nakai elephant population.

The Convention on Biological Diversity (UNEP, 1992) defined genetic diversity as one of the three pillars of biodiversity (Hoban et al., 2021) as it provides the raw material on which natural selection acts. In the early stages of population reduction and fragmentation, diversity in the form of rare alleles may be lost and with them the heritable variation needed to adapt to environmental changes, including changes in climate and the emergence of novel pathogens (Wernberg et al., 2018). As population numbers decline, alleles continue to be lost through random genetic drift. If this decline is accompanied by barriers to gene flow, inbreeding can further erode the ability of a population to maintain viability over the long term (Frankham, 2022). Thus, monitoring the levels of genetic diversity is an essential component of population management (Hoban et al., 2021).

The Nakai Plateau underwent a major habitat transformation following the completion of the NT2 hydroelectric dam in April 2008. Prior to dam completion, most of what became the 450 km^2^ Nam Theun reservoir was forested, and an estimated 40% of suitable elephant habitat on the plateau was lost as a result (McWilliam et al., 2010). Here, we combine the results of studies of elephant presence, movements, and the incidence of HEC in the Nakai Plateau and surrounding areas before and for 12 years after dam completion with a genetic study of the Nakai elephant population 10 years after dam completion (Eggert & Ruiz‐Lopez, 2012). Our objectives were to (1) assess the geographic patterns of elephant presence and dispersal, mainly relating to human–elephant conflict (HEC), before and after the completion of the NT2 dam, (2) compare population size, demography, and levels of genetic diversity in the Nakai elephants before (Ahlering, Hedges, et al., 2011; Hedges et al., 2013) and 10 years after the completion of the dam, and (3) assess differences between the wet and dry seasons in the 2018/2019 Nakai elephant population to more fully understand contemporary movement patterns and population dynamics in the Nakai elephants.

## 2 METHODS

### 2.1 Geographic patterns of elephant presence and human‐elephant conflict

**FIGURE 1:**
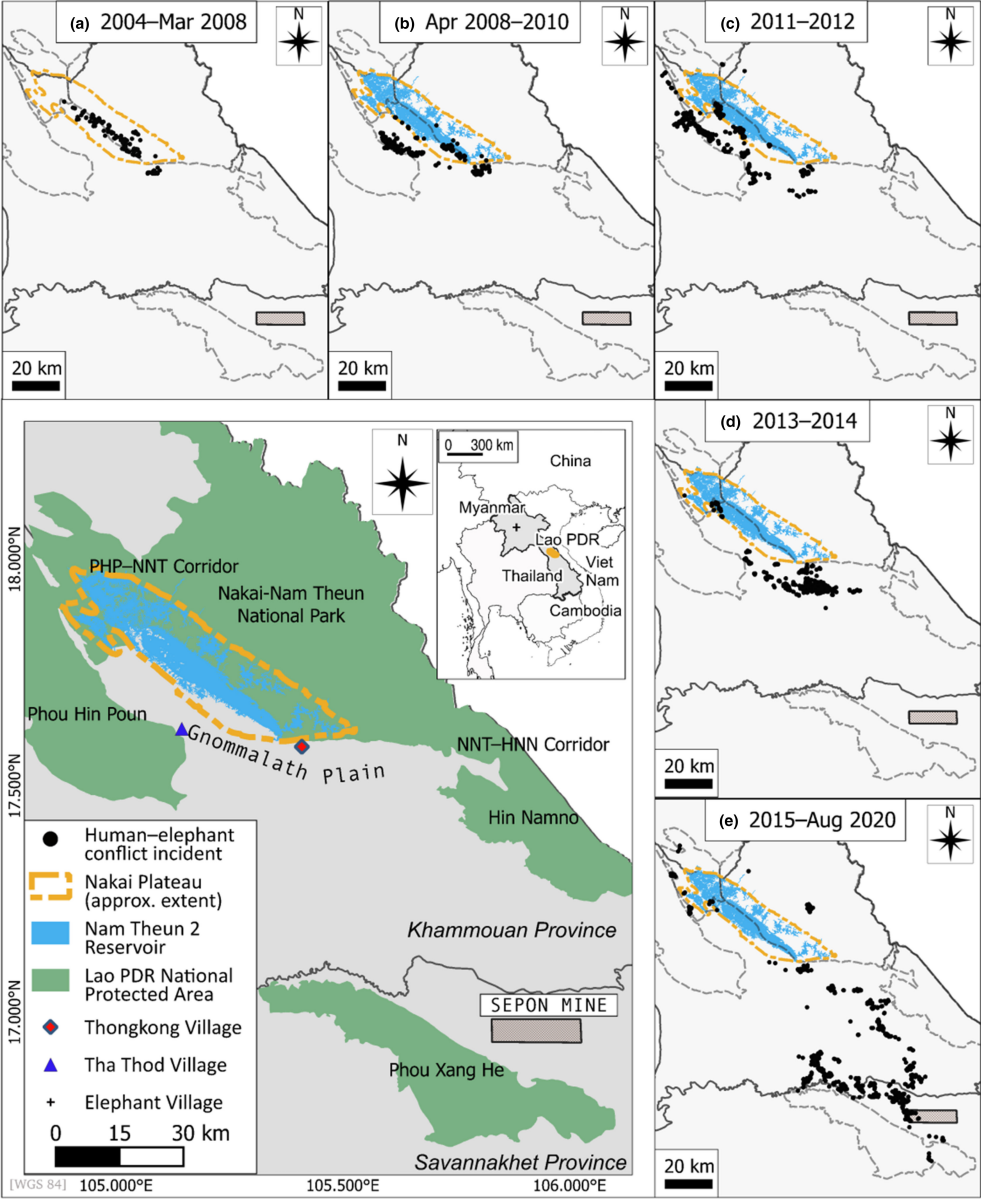
The Nakai plateau and surrounding landscape. (a) Human–elephant conflict (HEC) incident locations on the Nakai Plateau and in the surrounding landscape (pre‐inundation, October 2004–March 2008); (b) HEC incidents April 2008–December 2010; (c) HEC incidents January 2011–December 2012; (d) HEC incidents January 2013–December 2014; (e) HEC incidents January 2015–14 August 2020. (Data from: Hedges et al., 2007; McWilliam et al., 2010; Tyson & Phakphothong, 2015; and NTPC unpub. data).

Starting in October, 2004, prior to dam construction, several studies were conducted to better understand the dynamics of elephant populations and the incidence of HEC in the Nakai Plateau and surrounding areas (Hedges et al., 2007; McWilliam et al., 2010; Tyson & Phakphothong, 2015; Tyson & Rasphone, 2013; Tyson & Stremme, 2020). From October, 2004, to August, 2020, the Wildlife Conservation Society (WCS), in collaboration with the District Agriculture and Forestry Offices (DAFO) of affected districts, used monitoring teams to visit villages both on and off the Nakai Plateau (Figure 1) at least once a month in response to HEC reports. Beginning in 2009, a team from the Nam Theun 2 Power Company (NTPC) partnered with WCS and DAFO in these efforts. At each visit, monitoring teams recorded the GPS location of each HEC incident, any available details about the elephants involved such as sex, age and group size, and the types of damage incurred.

Between January and March 2011, a study focusing on the Sepon Mine area (Figure 1), some 100 km from the Nakai Plateau, was conducted in response to reports of increases in elephant presence and HEC (Hedges & Hallam, 2011). In addition to monitoring of HEC, teams collected dung samples using a Capture‐Mark‐Recapture (CMR) protocol (Hedges & Lawson, 2006) for a separate study designed to estimate population size and demography (Eggert & Ruiz‐Lopez, 2012).

In 2009, a group of three elephants moved away from the Nakai Plateau into the Gnommalath Plain area and regions southwest of the plateau, subsequently causing fear among villagers, crop‐raiding, and property damage in villages that had no previous history of HEC. The “group of three” (G3) elephants were routinely aggressive toward villagers, even during daylight (Tyson & Phakphothong, 2015; Tyson & Rasphone, 2013; Tyson & Stremme, 2020). By 2019, the group had grown to 6 individuals. Monitoring of movements and reports of HEC by these individuals was conducted by WCS/DAFO/NTPC teams as in the other regions near the Nakai Plateau.

### 2.2 Sampling for genetic analyses

To characterize the Nakai Plateau elephant population 10 years after dam completion, we collected fresh dung samples on the Nakai Plateau from 01 March 2018–01 May 2018 (dry season), and from 01 October 2019–01 November 2019 (wet season). Using the same approach as the 2006 survey (Hedges et al., 2013), we focused our efforts near wildlife trails and corridors, mineral licks, locations of recent sighting reports by local fishermen, and other known elephant “hotspots.” We conducted extensive boat‐based surveys to search for recent elephant damage to river and stream banks, and thus fresh elephant dung for collection, throughout the network of rivers within the boundaries of NNT NP. In April 2018, an additional 10 samples (9 adult females and 1 juvenile male) were collected from a captive population of retired timber elephants in Luang Prabang's Elephant Village in north‐central Laos (309 km from Nakai on a bearing of 309 degrees, Figure 1) to serve a known outgroup.

Fresh dung piles were identified using the criteria of Hedges and Lawson (2006). From each fresh dung pile, approximately 10 g of feces were preserved in 40 mL polypropylene tubes in Queen's College Buffer (QCB, Amos et al., 1992). The circumferences of the three largest boli in each dung pile were measured to provide an estimation of elephant age (Tyson et al., 2002). All samples were stored at −20°C prior to export to the Eggert lab at the University of Missouri for genetic analyses.

Samples from the 2018/19 survey were compared with those from the 2006 genetic CMR survey of the Nakai Plateau (Ahlering, Hedges, et al., 2011) and the 2011 genetic CMR survey of the Sepon mine/Phou Xang He NPA (PXH NPA) area (Eggert & Ruiz‐Lopez, 2012). Although the sequencing platform did not change between surveys, methods of amplification (singleplex vs. multiplex) and potential differences between allele calling by researchers make it difficult to compare studies (Ellis et al., 2011). Thus, we calibrated the results of those studies with the 2018/2019 study. DNA extractions from the 2006 Nakai Plateau survey had been preserved for long‐term storage at −80°C in elution buffer EB (Qiagen) and dung samples from the 2011 Sepon mine/PXH NPA survey (collected from January 13, 2011 to March 31, 2011) had been preserved in QCB and stored at −80°C. Since dung extractions contain DNA from not only the defecator, but also that of food items, parasites, and intestinal flora, we reasoned that samples with high DNA concentrations would be most likely to have retained enough elephant DNA for genetic analyses. Extractions from the Nakai 2006 study were concentrated to 20 μL using an ethanol precipitation and quantified in a NanoDrop spectrophotometer (Thermo‐Fisher Scientific). Based on a 260/280 ratio closest to 1.80 with a DNA concentration greater than 15 ng/μL, the 10 highest quality samples were selected for amplification. DNA was extracted from the samples from Nakai 2018/19, Elephant Village, and 10 samples from Sepon 2011 using a modified QIAamp DNA stool minikit protocol (Archie et al., 2008) that was optimized by Budd et al. (2021).

Permissions for sample collection were obtained from the Government of Lao PDR, the NNT NP, and the NT2 Power Company (NTPC). This study was conducted in compliance with Article 17, paragraph 2, of the Nagoya Protocol on Access and Benefit Sharing (Certificate #ABSCH‐IRCC‐LA‐256921‐1).

### 2.3 Genetic analyses of Nakai 2018/2019 samples

Samples were amplified at 18 multiplexed microsatellite loci (Eggert et al., 2000, 2008; Kongrit et al., 2008; Table 1). Amplification products were visualized in an agarose gel, purified, submitted for fragment analysis in an ABI 3730xl DNA analyzer (Thermo Fisher Scientific) with added 600 LIZ size standard at the University of Missouri DNA Core Facility and scored following Budd et al. (2021) in GeneMarker v.1.9.7 (Soft Genetics). To determine the sex of individuals, samples were amplified at two short Y‐specific fragments (SRY1 and AMELY2) and a longer X‐specific fragment (PLP1), following Ahlering, Hailer, et al. (2011).

**TABLE 1:** Microsatellite loci used to evaluate genetic diversity in the Nakai elephant population for the 2018 dry season (*N* = 58), 2019 wet season (*N* = 38), and all individuals (*n* = 91, five individuals were shared between seasons).

Locus	References	Label	Multiplex		NAKAI 2018			NAKAI 2019			NAKAI 2018 / 2019		
			A	B	H_O_	H_E_	*p*	H_O_	H_E_	*p*	H_O_	H_E_	*p*
EMU01	Kongrit et al. (2008)	VIC	3	2	0.636	0.662	.004	0.528	0.651	.000*	0.570	0.666	.000*
EMU02	Kongrit et al. (2008)	VIC	4	4	0.345	0.390	.001*	0.553	0.444	.486	0.420	0.413	.006
EMU03#	Kongrit et al. (2008)	6‐FAM	1	4	0.472	0.491	.007	0.371	0.431	.040	0.434	0.476	.002*
EMU04#	Kongrit et al. (2008)	NED	1	1	0.407	0.428	.006	0.444	0.518	.010	0.447	0.492	.000*
EMU06	Kongrit et al. (2008)	6‐FAM	3	2	0.545	0.671	.028	0.353	0.420	.012	0.500	0.625	.001*
EMU07#	Kongrit et al. (2008)	VIC	1	2	0.704	0.847	.000*	0.618	0.791	.006	0.663	0.835	.000*
EMU08	Kongrit et al. (2008)	VIC	3	1	0.328	0.428	.015	0.649	0.581	.013	0.444	0.498	.011
EMU09	Kongrit et al. (2008)	VIC	4	1	0.558	0.688	.001*	0.556	0.634	.068	0.554	0.679	.000*
EMU10#	Kongrit et al. (2008)	6‐FAM	1	2	0.608	0.555	.361	0.441	0.632	.001*	0.550	0.602	.001*
EMU11	Kongrit et al. (2008)	NED	4	4	0.789	0.721	.091	0.553	0.730	.003	0.700	0.732	.006
EMU12#	Kongrit et al. (2008)	VIC	2	3	0.411	0.634	.000*	0.486	0.610	.130	0.443	0.636	.000*
EMU13#	Kongrit et al. (2008)	NED	2	3	0.733	0.820	.052	0.667	0.781	.268	0.686	0.810	.002*
EMU14#	Kongrit et al. (2008)	6‐FAM	2	3	0.630	0.756	.016	0.829	0.791	.782	0.690	0.781	.111
EMU15#	Kongrit et al. (2008)	NED	2	4	0.604	0.651	.672	0.714	0.720	.445	0.639	0.676	.891
EMU17#	Kongrit et al. (2008)	NED	3	3	0.809	0.787	.286	0.714	0.822	.005	0.757	0.805	.023
EMU18	Kongrit et al. (2008)	6‐FAM	4	1	0.630	0.626	.108	0.568	0.636	.020	0.593	0.630	.002*
LA4	Eggert et al. (2000)	NED	5	2	0.417	0.738	.000*	0.387	0.770	.000*	0.392	0.760	.000*
FH94R	Eggert et al. (2008)	NED	5	4	0.340	0.576	.000*	0.441	0.691	.000*	0.377	0.640	.000*
Mean					0.554	0.637		0.548	0.647		0.548	0.653	
SE					0.036	0.032		0.031	0.03		0.028	0.029	

*Note*: The nine loci used in genetic comparisons of Nakai 2018, Nakai 2019, Nakai 2006, Sepon 2011, and the Elephant Village are indicated by #. Label = fluorescent label used on forward primer; Multiplex = reaction number within combination A or B; H_O_ = observed heterozygosity; H_E_ = expected heterozygosity under Hardy–Weinberg equilibrium (HWE); *p* = deviance from expected value under HWE with * designating significance following Bonferroni correction.

To quantify the minimum number of matching loci needed to identify individuals, genotypes were analyzed for probability of identity (pID) and probability of identity for siblings (pSibs) in GenAlEx v.6.41 (Peakall & Smouse, 2012) using the thresholds set by Waits et al. (2001; pID < .001; pSibs < .01). We determined unique genotypes and recaptures in Cervus v.3.0.7 (Kalinowski et al., 2007) using the “fuzzy matching” function to identify genotypes that matched at all but three alleles.

We tested for Hardy–Weinberg equilibrium and linkage disequilibrium in Genepop on the Web (Raymond & Rousset, 1995; Rousset, 2008) for 2018 and 2019 independently and together. We tested for population closure overall and by sampling season in program Capture (Otis et al., 1978; White et al., 1982; software accessed 10/2021 at https://www.mbr‐pwrc.usgs.gov/software/capture.html). Per locus diversity was estimated as observed heterozygosity (H_O_), expected heterozygosity (H_E_), Shannon's information index (I), and the mean fixation index/inbreeding coefficient (*F*_IS_) in GenAlEx v.6.41, and allelic richness (A_R_) and private allelic richness (A_P_) in HP‐Rare v. 1.1 (Kalinowski, 2005). We tested for relatedness between all females and young (dung bolus circumference < 30 cm) and all adult and subadult males were detected near each other within a 48 h period using Goodnight and Queller (1999) in GenAlEx v.6.41.

We tested for genetic differentiation between seasons using *F*_ST_ and assessed significance levels using 9999 permutations in GenAlEx v.6.41. For individuals with a pairwise relationship of *r* > .25, one individual from the pairing was removed prior to the inference of genetic population structure using Structure v.2.3.4 (Pritchard et al., 2000) for up to 10 clusters (K) with 10 replicates for each K. We ran 50,000 burn‐in steps and 250,000 MCMC replicates using an admixture model with no priors. We determined the best‐supported number of genetic clusters using the ∆K method of Evanno et al. (2005) in Structure Harvester v.0.6.94 (Earl & VonHoldt, 2012).

For each unique genotype, we amplified an approximately 630 bp fragment of mitochondrial DNA (mtDNA) containing a portion of the C terminal of cytochrome *b*, the threonine and proline tRNAs, and the 5′ end of the noncoding control region (d‐loop), using primers MDL3 and MDL5 (Fernando et al., 2000) and the conditions outlined in Eggert et al. (2002). We sequenced amplification products in both directions in an ABI 3730xl DNA Analyzer (Thermo Fisher Scientific) and aligned and edited sequences in Geneious v. 8.0.5 (Kearse et al., 2012). Haplotypes were collapsed using FaBox v.1.41 (Villesen, 2007) and compared to those in GenBank for sequence similarity.

### 2.4 Genetic and demographic comparisons of Nakai 2018/2019, Nakai 2006, and Sepon 2011

Ten selected samples from the Nakai 2006 study and nine samples from the Sepon 2011 study were amplified and re‐genotyped at the nine Asian elephant loci used in the Nakai 2006 survey (Ahlering, Hedges, et al., 2011; Table 1). The remaining genotypes produced during the Nakai 2006 and Sepon 2011 studies were calibrated at each of the nine loci using the results from the re‐genotyped individuals.

Per locus diversity was calculated as shown for the 2018 and 2019 Nakai samples. We tested for the normality of each per locus metric using a Shapiro‐Wilks' test for normality and arcsine square root transformed the per locus H_E_ and H_O_ in RStudio v.3.5.2 (R Core Team, 2018). Significant differences in diversity between populations were determined using general linear models with locus as a fixed effect using the Lme4 package v.1.1‐23 (Bates et al., 2015) with A_R_ and A_P_ transformed to gamma distributions. These tests were followed by an ANOVA in the package car v.3.0‐9 (Fox & Weisberg, 2019) and post‐hoc testing using Tukey tests in multcomp v.1.4‐13 (Hothorn et al., 2008).

Comparison of the proportion of the total genetic variance contained within each population relative to the total genetic variance (*F*_ST_) was performed in GenAlEx v.6.41 using 9999 permutations with a standard Bonferroni correction to determine statistical significance (Neyman & Pearson, 1928). Genetic population structure across sites and studies was inferred in Structure v.2.3.4 (Pritchard et al., 2000) using the methods described for the 2018/2019 samples.

For mtDNA, we calculated haplotypic diversity (*h*) and nucleotide diversity (*π*) for Nakai 2018 and 2019 and added those results to the mtDNA results from the Nakai 2006 (Ahlering, Hedges, et al., 2011) and the Sepon 2011 (Eggert & Ruiz‐Lopez, 2012) studies. We determined significant differences using *genetic_diversity_diffs.R* v.1.0.6 (Alexander et al., 2016), a custom script that resamples from the combined haplotype frequencies overall populations and conducts a permutation test with 10,000 replicates. We tested for genetic differentiation (*ɸ*_ST_) in Arlequin v.3.5.1.2 (Excoffier et al., 2005; Excoffier & Lischer, 2010), assessing the significance of differentiation using 1000 permutations and applying a standard Bonferroni correction (Neyman & Pearson, 1928).

We assessed demographic differences between Nakai 2018 (dry season), Nakai 2019 (wet season), and Nakai 2006 (dry season) using fecal bolus circumferences as a proxy for elephant age (Tyson et al., 2002). We tested for deviations from the normality of distributions using a Shapiro‐Wilks' test for normality; after finding that the distribution of females in the Nakai 2006 dataset was not normal, we conducted a Mann–Whitney test in Stats v.4.0.3 (R Core Team, 2018) to assess differences.

## 3 RESULTS

### 3.1 Geographic patterns of elephant presence and HEC

In Phase 1 of the Elephant Program (October 2004 to May 2006), baseline assessments of HEC covered 14 villages on the Nakai Plateau and two villages in the Thongkong area (Gnommolath district). A relatively low level of HEC occurred (Figure 1a, Table 2), with an average of 6.95 incidents/month, mainly involving damage to crops and limited damage to huts and livestock sheds (Hedges et al., 2007). In Phase 1.5 (July 2006 to May 2007), the monthly rate of HEC was similar (average of 10.09 incidents/month). However, during Phase 1.5, while incidents continued to be focused mainly on trampling and raiding of crops, they were concentrated in 6 villages as compared to 16 villages during Phase 1. Phase 2 of the Program (June 2007 to December 2009) included the period of impoundment creating the reservoir. During this Phase, the average rate of HEC increased to an average of 18.03 incidents/month involving 30 villages (McWilliam et al., 2010). Post‐April 2008, when the reservoir began to fill, the rate was 22.2 incidents/month. Of the 30 villages, 16 in the Thongkong and Tha Thod areas (Figure 1b) experienced HEC for the first time since monitoring began in 2004. Although rice, cassava, banana, and coconut continued to be the most commonly damaged or destroyed crops, new targets were recorded during this period including harvested rice stored in villagers' houses and cotton. Farmers also reported that elephants killed three buffaloes and three goats; however, monitoring teams were unable to find direct evidence that these deaths were caused by elephants (McWilliam et al., 2010).

**TABLE 2:** Human–elephant conflict (HEC) incidents and number of villages affected (in parentheses), by area, prior to and post‐completion of the dam creating the NT2 reservoir (sources: Hedges et al., 2007; McWilliam et al., 2010, and NTPC, unpublished).

Years	No. of months	Nakai plateau/NNT NP	Thongkong area	Tha Thod area
Prior to NNT2 dam completion				
October 2004–May 2006	20	120	19	0
July 2006–May 2007	11	85	26	0
June 2007–April 2008	11	77	39	0
Post dam completion				Tha Thod area & Off‐Plateau
May 2008–December 2009	20	169 (8)	123 (9)	151 (13)
2010	12	21 (5)	130 (2)	164 (9)
2011	12	39 (4)	81 (1)	349 (20)
2012	12	229 (11)	30 (2)	281 (37)
2013	12	64 (6)	47 (1)	524 (34)
2014	12	1 (1)	49 (3)	314 (31)
2015	12	19 (4)	9 (1)	455 (53)
2016	12	0	7 (1)	50 (13)
2017	12	3 (1)	15 (3)	37 (11)
2018	12	1 (1)	6 (1)	27 (3)
2019	12	1 (1)	0 (0)	88 (13)
January 2020–August 2020	8	3 (1)	0 (0)	124 (19)

Once the reservoir reached full level in mid‐2009, HEC in Plateau areas was reduced while there was an increase in the number of incidents off the Plateau, both in the Thongkong area, where the number of villages affected as well as the number of incidents increased, especially in the 2009–2012 period, and in the Tha Thod area (Figure 1b,c; Table 2).

HEC caused by elephants likely dispersing from the Nakai Plateau also occurred across the wider landscape (Figure 1c). During fieldwork by WCS staff in and around the Sepon mine area, some 100 km from the Nakai Plateau, monitoring teams were told by farmers that HEC had increased over the previous years.

Monitoring of the G3 elephants indicated that they had extended their range to the south and east of that recorded in 2013 (Figure 1d,e). In 2016, the G3 elephants moved into the PXH NPA, approximately 100 km southeast of the Nakai Plateau in Savannakhet Province. In January 2018 and in November/December 2019 (Tyson & Stremme, 2020), attempts to immobilize and fit at least one of the G3 elephants with a satellite collar to enhance monitoring efforts were unsuccessful.

### 3.2 Genetic analyses of Nakai 2018/2019 samples

We collected 125 fresh dung samples from the Nakai Plateau: 84 samples during the dry season in 2018 and 41 during the wet season in 2019. Analysis of a preliminary subset of samples determined that genotypes from a minimum of eight microsatellite loci were needed to have a high probability of identifying individuals (pID = .000; pSibs = .005). Two samples failed to amplify at the required minimum number of eight loci and were removed from further analyses. From the 123 remaining samples, we detected 91 unique genotypes/individuals; 53 from 2018, 33 from 2019, and five captured during both seasons. We recaptured 15 individuals, and within those, we had a mean recapture rate of 3.133 ± 0.957. Program Capture (Otis et al., 1978; White et al., 1982) determined a lack of population closure overall and for each season. Therefore, we were unable to conduct further analyses of population size through the use of standard CMR methods.

Despite all 18 loci being polymorphic with no evidence of linkage disequilibrium, analyses that combined individuals from wet and dry seasons found that 12 of the 18 loci did not conform to expectations under Hardy–Weinberg equilibrium (Table 1). Further analysis in GenAlEx suggested significant genetic differentiation between seasons (*F*_ST_ = 0.012; *p* = .007). Thus, we chose to separate the data by season in all further analyses. After separation, only EMU07 and EMU12 did not conform to HWE expectations in 2018 and EMU10 did not conform in 2019. There were no departures from expectations in the Sepon data, and we did not test for departures in the Elephant Village dataset since it was made up of 10 retired timber elephants and does not represent a natural, random mating population. Because there were no consistent departures from expectations under HWE across populations, we retained all nine loci in our analyses.

In Nakai 2018 (dry season), we found 15 adults, 27 subadults, 11 juveniles, corresponding to 34 females and 19 males. In Nakai 2019 (wet season), we found nine adults, 12 subadults, and seven juveniles, representing 19 females and nine males. We also found four females and one male during the wet season whose ages could not be determined due to a lack of intact dung boli. The five individuals found in both seasons consisted of an adult female, two subadult females, and two subadult males. The average pairwise Goodnight and Queller relatedness between females and juveniles was −0.002 (SE 0.022, 95% CI −0.161 to 0.152) while males were—0.037 (SE 0.093, 95% CI −0.348 to 0.299).

### 3.3 Comparisons between the Nakai studies of 2006, 2018, and 2019

We successfully re‐genotyped 10 individuals from Nakai 2006 and nine individuals from Sepon 2011, allowing for a per‐locus calibration with the 2018 and 2019 Nakai data. Our final calibrated datasets for population comparisons included 58 individuals from the Nakai 2018 dry season, 38 from the Nakai 2019 wet season (we included the 5 individuals captured in both seasons in each count), 10 from the Elephant Village, 31 from the 2011 Sepon survey, and 102 from the Nakai 2006 dry season survey at the nine shared microsatellite loci and the mtDNA fragment.

We found significant declines in nuclear microsatellite diversity from Nakai 2006 to Nakai 2018 and 2019, in expected heterozygosity (Table 3; Nakai 2006 vs. Nakai 2018 dry season *p* = .011, Nakai 2006 vs. Nakai 2019 wet season *p* = .039) and Shannon's information index (Nakai 2006 vs. Nakai 2018 dry season *p* = .005, Nakai 2006 vs. Nakai 2019 wet season *p* = .028). We also found a significant difference in allelic richness between the Nakai 2006 population and the Nakai 2019 populations (ANOVA *p* = .007; Nakai 2006 vs. Nakai 2019 wet season *p* = .022) and between the Nakai 2018 dry season and 2019 wet season (*p* = .010).

**TABLE 3:** Diversity values (±SE) for the nine microsatellite loci shared among studies in Nakai 2006, Sepon 2011, Nakai 2018, and Nakai 2019 including observed heterozygosity (H_O_), expected heterozygosity (H_E_), fixation index (*F*_IS_), Shannon's index (I), allelic richness (A_R_), and private allelic richness (A_P_).

	*N*	H_O_	H_E_	*F*_IS_	I	A_R_	A_P_
Nakai 2006	102	0.667 ± 0.037	0.745 ± 0.028	0.106 ± 0.035	1.633 ± 0.105	7.646 ± 0.559	0.961 ± 0.260
Sepon 2011	31	0.566 ± 0.053	0.555 ± 0.045	−0.019 ± 0.042	1.003 ± 0.092	5.534 ± 0.494	0.164 ± 0.092
Nakai 2018	58	0.597 ± 0.047	0.663 ± 0.050	0.093 ± 0.043	1.389 ± 0.142	7.756 ± 0.851	0.783 ± 0.194
Nakai 2019	38	0.587 ± 0.052	0.677 ± 0.046	0.138 ± 0.035	1.432 ± 0.120	6.639 ± 0.438	0.283 ± 0.126

**FIGURE 2:**
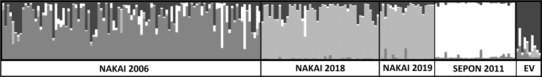
Population structure results from Structure 2.3.4 based on 9 nuclear microsatellite loci for K = 4 (∆K = 39.42) with cluster 1 in medium gray, cluster 2 in light gray, cluster 3 in white, and cluster 4 in dark gray (EV = Elephant Village).

We identified 64 individuals that had at least one relative at the level of *r* > .25, with 31 having at least 1 relative at the *r* ≥ .50. From these individuals, we identified 13 relative groups united by probable parent‐offspring, full‐sibling, or half‐sibling relationships. Nine of these relative groups consisted of only two full‐sibling individuals. The largest relative group was united through an adult male that had 17 relatives with *r* > .125 (cousin), 13 of which were *r* > .25 < .50 (sibling or half‐sibling). This male has likely fathered at least three individuals (*r* ≥ .5). Analysis of the data in Structure v.2.3.4 identified four genetic clusters overall (K = 4; ∆K = 39.42). These were broadly defined as Cluster 1—Nakai 2006, Cluster 2—Nakai 2018 and Nakai 2019, and Cluster 3—Sepon 2011 and Cluster 4—the Elephant Village (Figure 2). A majority of elephants in Nakai 2018 and 2019 assigned (Q ≥ 0.50) to Cluster 2, which was detected at low frequency in Nakai 2006. We found fewer individuals being assigned to Cluster 3 in Nakai 2018 than Nakai 2006, and none in Nakai 2019. Pairwise comparisons (*F*_ST_) based on the nine nuclear microsatellite loci shared among the studies found that all populations except Sepon 2011 and the Elephant Village were significantly different (Table 4).

**TABLE 4:** Genetic differentiation based on the nine nuclear microsatellite loci shared among studies (*F*_ST_) below diagonal and mtDNA (*ɸ*_ST_) above diagonal; significance following Bonferroni correction is indicated by *.

	Nakai 2018	Nakai 2019	Nakai 2006	Sepon 2011	Elephant Village
Nakai 2018	–	0.253	0.072*	0.304*	0.044
Nakai 2019	0.012*	–	0.024	0.181*	0.156
Nakai 2006	0.012*	0.026*	–	0.175*	0.230*
Sepon 2011	0.037*	0.043*	0.033*	–	0.541*
Elephant Village	0.059*	0.065*	0.056*	0.024	–

For mtDNA, in 2018 and 2019, we found five of the six previously discovered haplotypes from the Nakai Plateau (Table 5). Haplotypes LaoPDR‐A, LaoPDR‐B, LaoPDR‐C, and LaoPDR‐D (GenBank Acc# HQ113847–HQ113850) are from the Asian elephant α‐clade, whereas LaoPDR‐E and LaoPDR‐F (GenBank Acc# HQ113851 and HQ113852) are from the β‐clade (Fernando et al., 2000). LaoPDR‐A corresponds to AH of Vidya et al. (2009), LaoPDR‐D to AC (Vidya et al., 2009), and LaoPDR‐E to NewB haplotype in Thongchai et al. (2011). We found significantly higher nucleotide diversity in Nakai 2018 (*π* = 0.018 ± 0.009) than in Nakai 2006 (*π* = 0.011 ± 0.006, *p* = .001). This was largely driven by an increase in the frequency of haplotype LaoPDR‐E and the corresponding proportion of β clade haplotypes from 17% in 2006 to 47% in 2018 (Table 5). Based on mtDNA *ɸ*_ST_, Nakai 2006 significantly differed from Nakai 2018 (*p* = .000), but not Nakai 2019. Sepon 2011, which was found to have only 2 haplotypes, LaoPDR‐A and EmaxBN (GenBank Acc# AY245826, Vidya et al., 2009), differed from all other populations (Table 4).

**TABLE 5:** Mitochondrial haplotype frequencies for Nakai 2006, Sepon 2011, Nakai 2018, and Nakai 2019 and ratios of Alpha and Beta clades; GenBank accession numbers for each haplotype are shown in parentheses.

	α clade				β clade			α:β
	LaoPDR‐A (HQ113847)	LaoPDR‐B (HQ113848)	LaoPDR‐C (HQ113849)	LaoPDR‐D (HQ113850)	LaoPDR‐E (HQ113851)	LaoPDR‐F (HQ113852)	Emax‐BN (AY245826)	
Nakai 2006	39%	21%	1%	22%	16%	1%	0	83%:17%
Sepon 2011	71%	0	0	0	0	0	29%	71%:29%
Nakai 2018	24%	9%	3%	17%	47%	0	0	53%:47%
Nakai 2019	39%	8%	13%	11%	29%	0	0	71%:29%

**FIGURE 3:**
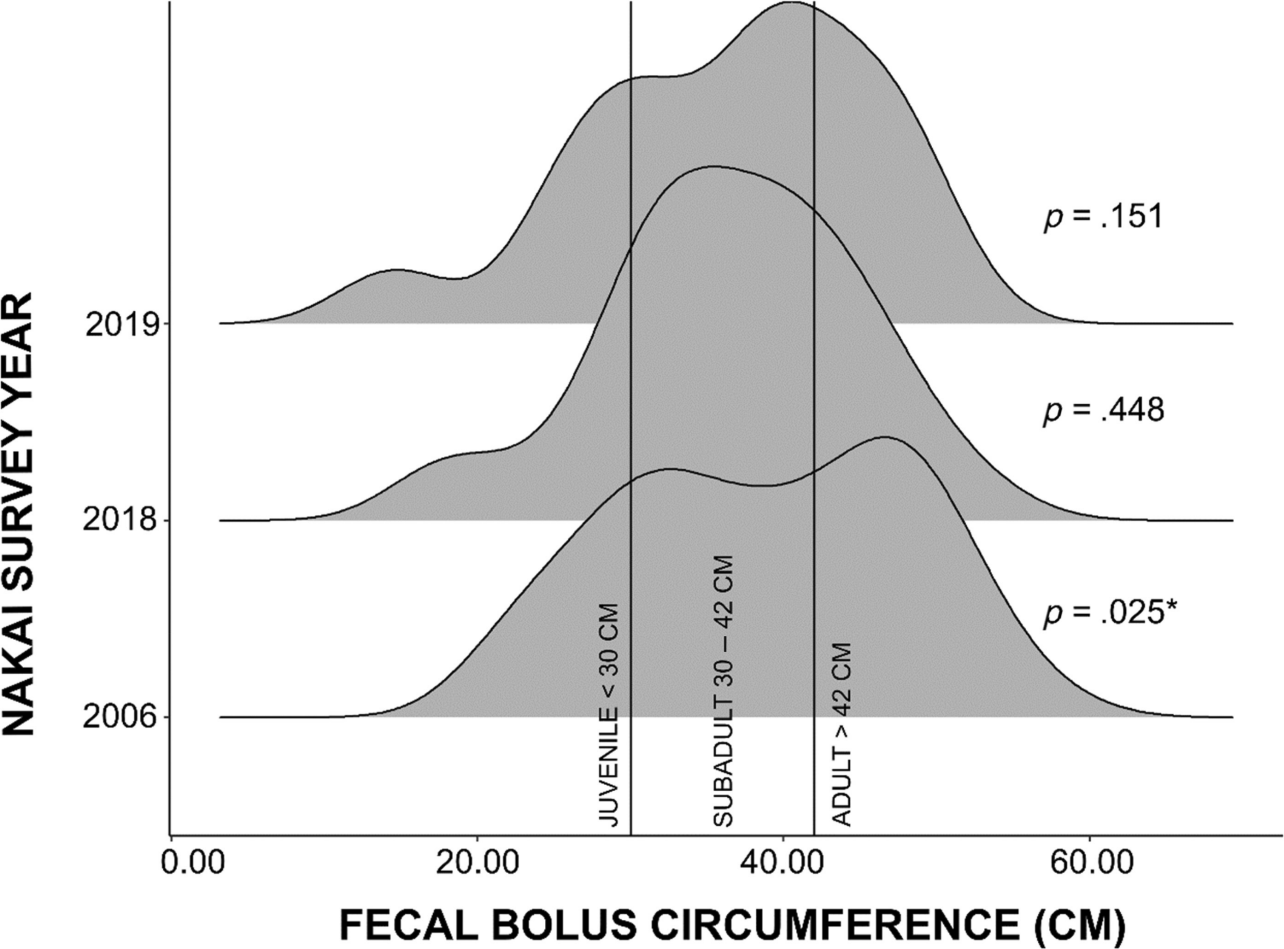
Age distribution density plots for the Nakai 2006, 2018, and 2019 surveys using fecal bolus circumference as a proxy for age; age class thresholds defined by Tyson et al. (2002) are indicated and significant differences are shown by *.

We found the Nakai 2018 and 2019 populations, combined and separately, to be normally distributed for fecal bolus circumferences (a proxy for age), while the Nakai 2006 dry season population had a bimodal distribution (*W* = 0.968, *p* = .025; Figure 3) that was driven by the non‐normality of females (*W* = 0.932, *p* = .046). Despite the differences in normality, Mann–Whitney tests between populations were non‐significant. There was a shift in the sex ratio of the population, with the ratio of males to females changing from 1:3 in Nakai 2006 to 1:2 ratio in Nakai 2018 and Nakai 2019.

## 4 DISCUSSION

The building of the NT2 hydroelectric dam and subsequent inundation of the Nakai Plateau provided a unique opportunity to study the effects of habitat transformation on an elephant population by monitoring movements and collecting data on HEC pre‐ and post‐inundation, both in the Nakai Plateau area and the wider landscape. The loss of high‐quality habitat for the Nakai elephant population corresponded with a major increase in HEC, and long‐range dispersal by some of the Nakai elephants likely fueled the creation of new, serious HEC problems across the wider landscape up to 100 km from the Nakai Plateau.

Prior to the period of flooding, the relatively low level of HEC detected was concentrated on the Nakai Plateau and in the Thongkong area (Figure 1a). From the beginning of flooding until the reservoir reached a full level in mid‐2009, villagers continued to opportunistically plant rice in areas that were not yet fully inundated (Tyson & Rasphone, 2013). In 2009 and 2010, HEC incident reports increased substantially (Table 2) and included the first reports from the Tha Thod area (Figure 1b). In 2011–2012, incidences of HEC continued to increase and were reported from areas to the west and northwest of the Plateau (Figure 1c). The period from 2013 to 2014 saw substantial increases in incidences to the south. Although there is no direct evidence that the elephants involved in HEC in these areas were from the Nakai Plateau, no other elephant populations were known to occur previously in these surrounding districts. Moreover, many farmers commented that the elephants involved in these incidents came from the Nakai Plateau using several tracks to descend off the plateau including an old logging road (McWilliam et al., 2010).

By 2015, HEC was frequently recorded to the south of the Plateau including the Sepon mines area. Local authorities attributed the increase in incidents to several factors, including an increase in disturbance in the Phou Xang He NPA (PXH NPA) involving logging and illegal killing of elephants, conversion of saltlicks to rice fields, an increase in the human population in the Sepon area, disturbance caused by the Sepon mine, and the inundation of the Nakai Plateau, which villagers said caused elephants to move off the Plateau and into the Sepon area. The reported timing of this last phenomenon coincided with the inundation of the Nakai Plateau in April 2008 (Hedges & Hallam, 2011).

The G3 elephants became a particular problem, as they displayed little fear of humans, had become habituated to living in areas with villages and agriculture (Tyson & Rasphone, 2013), and were involved in at least one human death (Tyson & Phakphothong, 2015). This contrasts with the PXH NPA elephants living adjacent to the Sepon mine area which also caused HEC, but avoided contact with people, based on radio‐collaring data (NTPC, unpublished), despite their very close proximity. Because attempts in January 2018 and November/December 2019 to radio‐collar at least one of the G3 elephants (Tyson & Stremme, 2020) were unsuccessful, the ability to detect their movements and activities continues to be limited.

Since demographic and genetic changes happen over longer timeframes than the continuous changes in elephant presence and HEC we detected, we examined them 10 years after dam completion during both the 2018 wet season and the 2019 dry season. For both the 2018 and 2019 data, we found a lack of population closure (necessary for standard CMR analysis, which assumes neither immigration nor emigration occurs during the survey period), with significant genetic differentiation between survey seasons and only five individuals shared between surveys. Ahlering, Hedges, et al. (2011) speculated that the Nakai Plateau elephants could be part of a larger metapopulation because of the unusually high levels of genetic diversity detected. Specifically, they suggested the possibility that rather than being an established resident population, the Nakai elephants might contain remnants of other populations that had been forced to relocate to the Nakai Plateau from other parts of Laos and Vietnam. Our 2018/2019 results neither support nor contradict that speculation but do suggest movements of elephants within the region as well as substantial differences in seasonal usage among individuals in the present Nakai population.

While our genetic results are based on a relatively small number of samples, they represent an approximately equal sampling effort between the 2006 and 2018/2019 surveys, each of which lasted approximately 90 days and involved multiple field teams. Given that, our data suggest that since dam completion, which resulted in the loss of approximately 40% of elephant habitat, there have been substantial changes in the population including a possible overall reduction in numbers, a change in the sex ratio, and a shift in the age structure to a higher proportion of sub‐adults. With regard to population sizes, a naive comparison of the 102 individuals known to be in the population in the 2006 dry season (i.e., the number of unique genotypes found) to the 58 found in the 2018 dry season suggests a severe reduction in population size. However, we caution that if the 2018 dry season and the 2019 wet season results are combined, a minimum of 96 elephants were found to be using the Nakai Plateau area in 2018 and 2019. Comparable survey data for the 2006 and 2007 wet seasons are not available, so it is unknown whether the population estimate might have been larger if elephant usage of the area in both of those seasons had been assessed. Further work on the population sizes, connectivity, and metapopulation dynamics of the elephant populations throughout the Bolikhamxay, Khammouane, and Savannakhet Provinces should be conducted to better understand their status, conservation significance, and management needs, particularly given continuing infrastructure development in these areas.

Over the 10 years between genetic surveys, there has been a shift in sex structure as the ratio of males to females was 1:3 in the 2006 dry season but 1:2 in both the 2018 dry season and the 2019 wet season. In Asian elephants, males are typically the dispersing sex while females exhibit philopatry (Vidya & Sukumar, 2005), therefore, this shift toward an increased proportion of subadult males may be indicative of bachelor dispersers investigating the Plateau. It could also result from a reduction in female‐biased philopatry in the area, as evidenced by the reduction in relatedness between females and juveniles as compared to the Nakai 2006 study. Furthermore, the non‐genetic data related to the G3 elephants showed female dispersal and thus demonstrated breeding females leaving the Nakai Plateau after inundation and establishing new home ranges in the PXH NPA area, some 100 km away.

We also detected a shift in the age structure of the population to a higher proportion of sub‐adults. In 2018 and 2019, populations were comprised of a relatively large number of subadults representing 53% and 42% of the population in the 2018 dry season and the 2019 wet season, respectively. In the 2006 dry season survey, subadults accounted for only 31% of the population.

The loss of nuclear genetic diversity following the construction of the NT2 dam is concerning, as the loss of diversity can affect long‐term population viability (Reed & Frankham, 2003). The average age of first reproduction in Asian elephants is 13 years (De Silva et al., 2013). Since our study was conducted 10 years after dam completion, it was below the generation time of elephants. Thus, the lower diversity levels likely reflect the effects of genetic drift in the smaller, more fragmented population that remains rather than the effects of reductions in gene flow due to the presence of the dam or inbreeding. While we did not detect a significant increase in inbreeding in 2018 and 2019 (as assessed using *F*_IS_ values; Table 3), the likely fewer individuals occupying the area in 2018 and 2019 could present future problems as generational turnover occurs. It is important to note, however, that the current level of nuclear genetic diversity exhibited by the Nakai Plateau elephant population, while lower than that detected in 2006, is comparable to other high‐diversity populations in Southeast Asia including those in Thailand (Kongrit et al., 2008), Cambodia (Gray et al., 2014), and Myanmar (Kusza et al., 2018).

In addition, the significantly lower allelic richness of 2019 compared to 2006 and 2018 was likely due to the number of close relatives found in the detected genotypes. We found a single male with 17 close relatives (.125 ≥ *r* < .50). Although this male likely only fathered three of those relatives, such high reproductive output by a few individuals in a small population can result in a rapid decline in effective population size (Caballero, 1994).

Having committed to a 23% increase in renewable energy by 2025 (ASEAN Centre for Energy, 2021), the countries of Southeast Asia face the challenge of developing the infrastructure needed. This will inevitably entail significant changes in land use to accommodate solar, wind, and hydropower. In their analysis of the potential for energy development in the region, Sakti et al. (2023) found that areas in northern Southeast Asia (Vietnam, Laos, Thailand, and the Philippines) had the highest potential for developing power from all three sources. The intention of Lao PDR to become the “battery of Southeast Asia” has resulted in over 50 hydroelectric dams in 15 years, with a further 101 under construction or planned (Chang, 2013; Williams, 2019). A study of existing and planned dams worldwide found that 1249 large dams are located in protected areas and that 509 new dams are currently planned (Thieme et al., 2020). While dams may benefit some species through, for instance, the creation of artificial wetlands, protected areas that contain dams cannot be considered to offer protection to the associated ecosystems (Sakti et al., 2023).

This study provides a greater understanding of not only the direct local impacts of hydropower projects on elephant populations, but also the landscape‐level effects of such projects, which to date have remained under‐appreciated and not included in mitigation plans. Future development of infrastructure projects should take into account potential landscape‐level (i.e., distant) impacts of altering key habitat for wide‐ranging species such as elephants, and should include assessments of potential human–wildlife conflict, including the economic impacts, and identify appropriate mitigation measures. The recent development of models that jointly consider habitat connectivity and human‐wildlife conflicts and guidelines on human‐wildlife conflict and coexistence (IUCN, 2023; Vasudev et al., 2023) are promising as they may provide a framework for conservation planning.

## AUTHOR CONTRIBUTIONS

**Kris Budd:** Conceptualization (equal); data curation (equal); formal analysis (equal); funding acquisition (equal); investigation (equal); methodology (equal); writing – original draft (equal). **Daophone Suddychan:** Investigation (supporting); writing – original draft (supporting); writing – review and editing (supporting). **Martin Tyson:** Data curation (equal); formal analysis (equal); investigation (equal); writing – original draft (equal); writing – review and editing (equal). **Camille N. Z. Coudrat:** Investigation (equal); writing – original draft (equal); writing – review and editing (equal). **Alex McWilliam:** Investigation (equal); writing – review and editing (equal). **Christopher D. Hallam:** Investigation (equal); methodology (equal); writing – review and editing (equal). **Arlyne Johnson:** Investigation (equal); writing – original draft (equal); writing – review and editing (equal). **Lori S. Eggert:** Conceptualization (equal); investigation (equal); methodology (equal); project administration (equal); writing – original draft (equal); writing – review and editing (equal).

## CONFLICT OF INTEREST STATEMENT

The authors declare that they have no conflicts of interest with respect to this work.

## Data Availability

Microsatellite genotype data has been deposited in DRYAD https://doi.org/10.5061/dryad.dv41ns247 and mitochondrial DNA sequence data is available on GenBank under the accession numbers provided.
