# Correction to “Effects of a hydropower project on a high‐value Asian elephant population”

**DOI:** 10.1002/ece3.10631

**Published:** 2023-11-28

Budd K, Suddychan D, Tyson M, Coudrat CNZ, McWilliam A, Hallam CD, Johnson A, Eggert LS. Effects of a hydropower project on a high‐value Asian elephant population. Ecology and Evolution 13(7): e10353, 2023, DOI: 10.1002/ece3.10353

Author S Hedges was not included. The correct author list should be:

Budd K, Suddychan D, Tyson M, Hedges S, Coudrat CNZ, McWilliam A, Hallam CD, Johnson A, Eggert LS.

We apologize for this error.

